# Influence of processing temperature on production of red beetroot powder as a natural red colorant using foam‐mat drying: Experimental and modeling study

**DOI:** 10.1002/fsn3.3621

**Published:** 2023-08-25

**Authors:** Golnaz Bahriye, Saeed Dadashi, Jalal Dehghannya, Hossein Ghaffari

**Affiliations:** ^1^ Department of Food Science and Technology University of Tabriz Tabriz Iran; ^2^ Department of Biosystems Engineering University of Tabriz Tabriz Iran

**Keywords:** drying, heat transfer, mass transfer, microstructure, modeling

## Abstract

With high medicinal and nutritional value, red beetroot powder is utilized as a natural pigment and functional additive in various food industries. In this investigation, red beetroot powder was produced using foam‐mat drying, and the effect of drying temperature on quality attributes was evaluated. Computer simulation was also performed to assess the impact of temperature on uniformity of moisture loss and temperature during drying. Temperature variations did not exert a significant impact on water solubility and total color difference of the powders. Images obtained from field emission scanning electron microscopy illustrated that with increasing temperature, wrinkling, cracking, and roughness of the powder particles reduced because of the formation of smooth and hard crusts on the particles' surface. Predicted moisture content values were in good agreement with measured data. The results of this study could be used to optimize foam‐mat drying of red beetroot to produce functional powders with suitable physicochemical and microstructural characteristics.

## Graphical abstract

Red beetroot powder was produced using foam‐mat drying as influenced by temperature. Coupled heat and mass transfer modeling was developed to optimize the drying process. Temperature rise improved morphology and some physicochemical properties of powders. The produced powder can be used as a safe replacement for artificial food colorants.

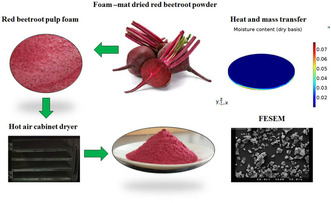

## 1 INTRODUCTION

Red beet (*Beta vulgaris* L. var. Detroit Dark Red) belongs to the *Chenopodiaceae* family produced in many temperate regions of the globe, particularly in Europe, Central America, North America, and Asia (Chhikara et al., 2019; Nistor et al., 2017). From a nutritional point of view, red beetroot has high levels of valuable healthy constituents such as fiber, essential vitamins, and vital minerals, as well as carotenoids, betalains, polyphenols, flavonoids, and saponins (Kohajdová et al., 2018). Because of their beneficial impacts on human health, organic red pigments, derived from fruits and vegetables, have drawn considerable attention as safe alternatives for synthetic red colorants in food industry (Deladino et al., 2017; Leong et al., 2018). The major natural pigments of red beetroot, responsible for its intense red color, are betalains (Bazaria & Kumar, 2016). They are water‐soluble nontoxic phytochemicals (Celli & Brooks, 2017; Leong et al., 2018; Sawicki et al., 2016), considered safe by FDA and EFSA, and are among the earliest natural colorants that have been used in food industry since 1967 (Calvo et al., 2018; Deladino et al., 2017; Khan, 2016; Pagano et al., 2018). Red beetroot is generally consumed in juice or powder form as a natural colorant and functional additive with beneficial properties for consumers, for example, in products such as dairy foods, tomato paste, sauces, marmalade, desserts, and beverages (Bazaria & Kumar, 2016; Chhikara et al., 2019; Kumar & Brooks, 2018; Ng & Sulaiman, 2018; Nistor et al., 2017). Besides its coloring capacity, it plays a number of significant roles in human health mainly thanks to its antioxidant, antimicrobial, and antitumor properties (Calvo et al., 2018; Chhikara et al., 2019; Kumar & Brooks, 2018; Leong et al., 2018; Rodriguez‐Amaya, 2019).

Dehydration is one of the most traditional techniques utilized to increase shelf life of perishable foods by eliminating moisture and preventing microbial spoilage. This is to enhance storage stability, to lessen postharvest losses of vegetables and fruits, and to reduce the costs of packaging, storage, and transportation because of the reductions in products weight and volume (Aamir & Boonsupthip, 2017; Castro et al., 2018; Horuz et al., 2017, 2018; İlter et al., 2018). Convective drying is the most common procedure applied to preserve food materials. Nevertheless, it has some drawbacks such as low drying rate, long processing time, and high‐energy consumption (Horuz et al., 2018; Kumar et al., 2015; Onwude et al., 2016; Wang et al., 2018; Zhang et al., 2017). Therefore, it is essential to improve the convective drying process and enhance quality of dried foodstuffs (Dehghannya et al., 2019; Zhao et al., 2014).

Foam‐mat drying technique has recently attracted attention of researchers owing to providing quick drying rates at lower drying temperatures, reducing drying time, and consequently acquiring better quality of dried products (Abbasi & Azizpour, 2016; Asokapandian et al., 2016; Franco et al., 2016; Ng & Sulaiman, 2018; Salahi et al., 2017). In this method, liquid or semiliquid substances are turned into a consistent foam by whipping and subsequently dried using various techniques such as convective, freeze, microwave, and vacuum drying (Abbasi & Azizpour, 2016; Azizpour et al., 2016; Franco et al., 2015). The quality of obtained powder relies not only on extrinsic drying conditions like velocity and temperature of air but also on intrinsic factors such as composition and thickness of foam (Franco et al., 2016).

Heat and mass transfer modeling can improve design of drying processes and optimize energy efficiency (Kumar et al., 2015; Malekjani & Jafari, 2018; Onwude et al., 2018, 2019; Perussello et al., 2014). Temperature and moisture profiles during drying can be predicted via computer simulation to control the process and enhance quality of dried products (Azzouz et al., 2018; Kumar et al., 2015). Azizpour et al. (2016) assessed influence of hot air drying temperature (45–90°C) on microstructure of foam‐mat‐dried shrimp puree. The results indicated that porosity of the product increased by increasing drying temperature. In another study, the impact of drying temperature (60 and 80°C) and concentration of foaming agent on qualitative characteristics of mango powder was investigated (Chaux‐Gutiérrez, Santos, et al., 2017). Temperature and concentration of foaming agent had no significant impact on solubility. However, the effective moisture diffusion coefficient indicated a significant ascending behavior with an increase in foaming agent concentration and drying air temperature. Dehghannya et al. (2019) mathematically modeled heat and mass transfer to investigate impact of convective hot air temperature on temperature and moisture distributions of lime juice during foam‐mat drying. Model validation represented a high correlation coefficient (>0.9) between experimental and model data. To the best of our knowledge, there is no study in the literature about simultaneous experimental and numerical study during drying of red beetroot as influenced by drying temperature. The aim of this study was to evaluate the impact of hot air temperature on physicochemical and microstructural properties during foam‐mat drying of red beetroot. In addition, heat and mass transfer modeling was performed to assess influence of temperature on heat and moisture loss profiles during drying.

## 2 MATERIALS AND METHODS

### 2.1 Material

Red beetroots (*Beta vulgaris* L. var. Detroit) were bought from a local vegetable market in Tabriz, Iran. Initial moisture content of the products was between 84% and 85% (wet basis). The red beetroots were washed, peeled, and sliced into small cubical pieces using a manual cutter. To limit enzymatic activity, blanching was performed in a water bath at 90°C for 7 min. Afterward, samples were rapidly immersed in ice water for 2 min (Paciulli et al., 2016). Blanched samples were ground up for 10 min until a homogenous pulp was achieved (Ng & Sulaiman, 2018). The obtained pulp was transferred into polyethylene (PE) zipper bags and kept at −18°C in a freezer until performing experiments (Chaux‐Gutiérrez, Pérez‐Monterroza, et al., 2017).

Ovalbumin powder as the foaming agent (A5253‐250G; Sigma‐Aldrich) was kept in refrigerator (4°C). Maltodextrin (Pooran Powder Sepahan, Isfahan, Iran) as the carrier agent with a dextrose equivalent (DE) value of 18–20 and methyl cellulose (M0512‐100G; Sigma‐Aldrich) as the stabilizing agent were stored at room temperature (25°C). Methyl cellulose solution was prepared by dissolving 5‐g methyl cellulose powder in 100‐mL distilled water and stirring by means of a magnetic mixer at 90°C until achieving a clear solution. The resulting solution was stored at 4°C for 24 h.

### 2.2 Preparation of foam

Red beetroot pulp was defrosted and ovalbumin (3% w/v), methyl cellulose solution (1% w/v), and maltodextrin (8% w/v) were added to the pulp. The resulting mixture was then whipped with an electric high‐speed blender (HM‐312, 300 W; Moulinex) at ambient temperature for 12 min (Ng & Sulaiman, 2018).

### 2.3 Drying procedure

**FIGURE 1:**
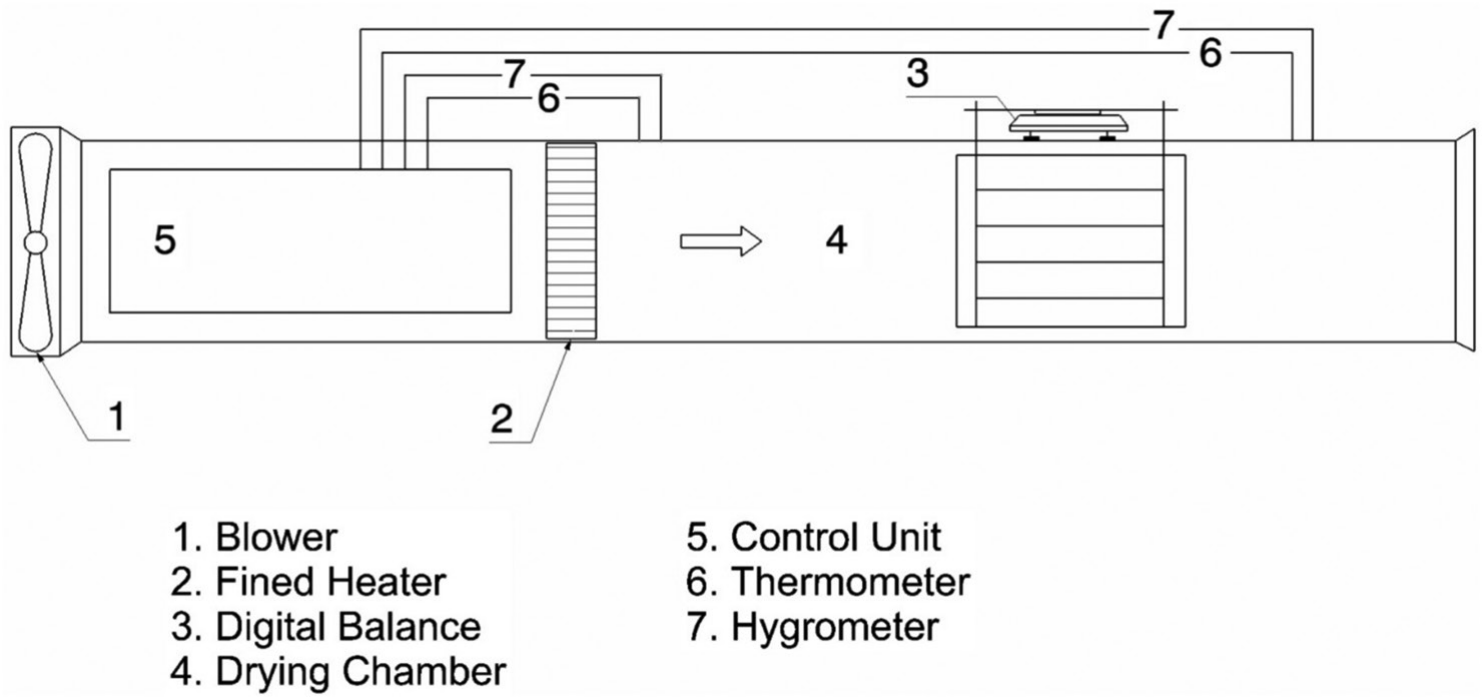
The convective dryer installation diagram.

A pilot‐scale cabinet‐type convective dryer with a chamber size of 2.37 × 0.345 × 0.345 m^3^ (length × width × height) fitted with six 5‐kW electric heaters, a thermometer, and two hygrometers at outlet and inlet was applied to dry beetroot foams (Figure 1). The foams were uniformly spread on specifically designed aluminum round plates with a diameter of 15 cm and thickness of 5 mm. Prior to drying, the dryer was run for about 30 min without sample to achieve the intended temperature. Drying tests were done at three different drying temperatures (50, 60, and 70°C). Hot‐air velocity was set to 1 m/s during all tests. Drying was carried out until the equilibrium moisture content was reached when there was no significant alteration in the weight of the samples between two consecutive moisture content measurements. All drying tests were performed in triplicate at each temperature. After drying, foam‐mat‐dried red beetroots were scraped off the plate using a spatula and ground in a crucible to obtain dry powders. The ground red beetroot powders were transferred to metalized polyethylene zipper bags and kept at 4°C until further analyses.

### 2.4 Drying kinetics

#### 2.4.1 Moisture ratio

Moisture ratio (MR) of the red beetroot foams during drying was calculated as (Khan et al., 2017):

(1) $$\mathrm{MR}=\frac{\left(X_{\left(t\right)}-X_{e}\right)}{\left(X_{i}-X_{e}\right)}$$

where *X*_(*t*)_ (db) is the moisture content (MC) at time *t*, *X*_*i*_ (db) is the MC at the start of drying, and *X*_*e*_ (db) is the equilibrium moisture content (EMC). Since EMC is very small compared to *X*_(*t*)_ and *X*_*i*_, the MR was calculated as (Khan et al., 2017):

(2) $$\mathrm{MR}=\frac{X_{\left(t\right)}}{X_{i}}$$

#### 2.4.2 Drying rate

Drying rate (DR) was determined as follows (Horuz et al., 2017):

(3) $$\mathrm{DR}=\frac{\left(X_{\left(t+\mathrm{\Delta t}\right)}-X_{\left(t\right)}\right)}{\mathrm{\Delta t}}$$

where *X*_(*t*+Δ*t*)_ (db) is the MC at time *t* + Δ*t*.

#### 2.4.3 Effective coefficient of moisture diffusivity (*D*_eff_)

*D*_eff_ was estimated using the Crank model for infinite slab (Crank, 1975; La Fuente et al., 2017):

(4) $$\mathrm{MR}=\frac{\left(X_{\left(t\right)}-X_{e}\right)}{\left(X_{i}-X_{e}\right)}=\frac{8}{\pi^{2}}\sum_{N=1}^{\infty }\frac{1}{{\left(1+2N\right)}^{2}}\exp\left(\frac{\left[-{\left(2N+1\right)}^{2}\pi^{2}D_{\mathrm{eff}}\right]}{4L^{2}}\right)t$$

where *X*_(*t*)_ (db) is the MC at time *t*, *X*_*i*_ (db) is the MC at the start of drying, and *X*_*e*_ (db) is EMC, *D*_eff_ (m^2^/s) is the moisture diffusion coefficient, *L* (m) is the foam thickness, and *t* (s) is the drying time. For lengthy drying operations, only the first term of Equation 4 is used:

(5) $$\mathrm{MR}=\frac{X_{\left(t\right)}}{X_{0}}=\frac{8}{\pi^{2}}\exp\left(-\frac{\pi^{2}D_{\mathrm{eff}}}{4L^{2}}t\right)$$

Taking the natural logarithm (ln) from both sides of Equation 5 results in:

(6) $$\ln\left(\mathrm{MR}\right)=\ln\left(\frac{8}{\pi^{2}}\right)+\left(-\frac{\pi^{2}D_{\mathrm{eff}}}{4L^{2}}\right)t$$

A straight line with negative slope is obtained by plotting the ln (MR) versus time. *D*_eff_ is calculated from the slope of the line (Olanipekun et al., 2015):

(7) $$\text{Slope}=-\frac{\pi^{2}D_{\mathrm{eff}}}{4L^{2}}$$

(8) $$D_{\mathrm{eff}}=\frac{\text{Slope}\times 4L^{2}}{\pi^{2}}$$

#### 2.4.4 Activation energy (*E*_*a*_)

The temperature dependency of *D*_eff_ was characterized by an Arrhenius‐type equation (Khan et al., 2017):

(9) $$D_{\mathrm{eff}}=D_{0}\exp\left(\frac{-\;E_{a}}{\mathrm{RT}}\right)$$

where *D*_0_ (m^2^/s) is the Arrhenius factor, *R* = 8.314 kJ/mol/K is the universal gas constant, *E*_*a*_ (J/mol) is the activation energy, *T* (*K*) is the absolute air temperature, and *D*_eff_ (m^2^/s) is the effective coefficient of moisture diffusion at *T*.

Taking the natural logarithm from both sides of Equation 9 leads to (Olanipekun et al., 2015):

(10) $$\ln D_{\mathrm{eff}}=\ln D_{0}+\left(\frac{-\;E_{a}}{\mathrm{RT}}\right)$$

*E*_*a*_ is calculated using the slope derived from the straight line from the graph of ln *D*_eff_ versus 1/*T* (Olanipekun et al., 2015):

(11) $$k_{2}=\frac{E_{a}}{R}$$

where *k*_2_ is the slope of the graph.

### 2.5 Analysis methods

#### 2.5.1 Bulk and tapped densities

Bulk (*ρ*_*B*,_ g/cm^3^) and tapped (*ρ*_*T*,_ g/cm^3^) densities of the powder were measured using the method of Asokapandian et al. (2016) with some modifications. Two grams of powder was slowly poured into a 10‐mL graduated glass cylinder. *ρ*_*B*_ was obtained by the ratio between the powder mass (g) and the volume occupied in the cylinder (cm^3^) without being tapped. Next, the graduated cylinder was tapped 10 times on a fabric from a height of 15 cm until a constant volume was reached. *ρ*_*T*_ was obtained by dividing the powder mass (g) by its final tapped volume (cm^3^).

#### 2.5.2 Particle density

Particle density ($\rho_{p}=\frac{M_{s}}{v_{s}}$, g/cm^3^) was obtained by the toluene displacement method (Dehghannya et al., 2019).

#### 2.5.3 Bulk porosity

Bulk porosity (*ε*, dimensionless) was calculated using the particle and bulk densities of the powder (Dehghannya et al., 2019):

(12) $$\varepsilon =1-\frac{\rho_{B}}{\rho_{P}}$$

#### 2.5.4 Flowability of powder

Carr index (CI, %) and Hausner ratio (HR, dimensionless) were calculated by (Seerangurayar et al., 2017):

(13) $$\mathrm{CI}=\frac{\rho_{T}-\rho_{B}}{\rho_{T}}\times 100$$

(14) $$\mathrm{HR}=\frac{\rho_{T}}{\rho_{B}}$$

Table 1 indicates the classification of powder flowability behavior, according to HR and CI (Seerangurayar et al., 2017).

**TABLE 1:** Classification of powder flowability according to Hausner ratio (HR) and Carr index (CI).

Flow property	HR	CI (%)
Very, very poor	>1.60	>38
Very poor	1.46–1.59	32–37
Poor	1.35–1.45	26–31
Passable	1.26–1.34	21–25
Fair	1.19–1.25	16–20
Good	1.12–1.18	11–15
Excellent	1–1.11	0–10

#### 2.5.5 Water absorption index (WAI) and water solubility index (WSI, %)

WAI and WSI (%) were obtained using the method of Asokapandian et al. (2016) with slight modifications. Two and a half gram of powder was carefully suspended in 30‐mL distilled water, and then, blended intermittently for 30 min using a magnetic stirrer at room temperature. The resulting solution was poured into a tube and centrifuged at 2071 × g for 5 min. After transferring the liquid supernatant into a preweighed plate, it was instantly dried in an oven at 105°C till a constant mass was reached. WAI was obtained as the mass (g) of the residual hydrated powder after centrifugation divided by the mass of the initial dry powder (g). Moreover, WSI (%) was calculated as the ratio of the dehydrated supernatant mass (g) to the initial dry sample mass (g).

#### 2.5.6 Moisture content (MC) of powder (%, w.b)

Powder samples were oven‐dried at 105°C for 24 h until reaching a constant mass. MC of the powders was calculated as (Seerangurayar et al., 2017):

(15) $$\mathrm{MC}\%=\frac{W_{1}-W_{2}}{W_{1}}\times 100$$

where *W*_1_ and *W*_2_ are the mass (g) of the powder before and after oven‐drying, respectively.

#### 2.5.7 Color evaluation

The powder sample was poured into a plate, and the surface was smoothened. Each powder sample was separately photographed by a digital camera (CANON, POWER SHOT S90, Japan) with a resolution of 10 megapixels in a white chamber under suitable lighting. Camera distance from the samples’ surface was 25 cm. Adobe Photoshop (CC 2017.1) was used to measure the L*, a*, and b* color parameters (Dehghannya et al., 2019). L*, varying between white (100) and black (0), represents lightness. a* and b* characterize redness and yellowness, respectively. The Δ*E* (total color difference), C* (Chroma or saturation index), *H*° (hue angle), and BI (browning index) were estimated using the L*, a*, and b* values (Aral & Beşe, 2016; Wang et al., 2018):

(16) ΔE=b0*−bt*2+a0*−at*2+L0*−Lt*212

(17) C*=bt*2+at*212

(18) $$H^{\circ }=\tan^{-1}\left(\frac{b_{\left(t\right)}^{*}}{a_{\left(t\right)}^{*}}\right)$$

where the subscripts 0 and *t* refer to color factors before and after drying, respectively.

(19) $$\mathrm{BI}=\frac{\left(-0.31+I\right)}{0.17}\times 100$$

where

(20) $$I=\frac{\left(1.75L^{*}+a^{*}\right)}{\left(-3.012b^{*}+a^{*}+5.645L^{*}\right)}$$

#### 2.5.8 Field emission scanning electron microscopy (FE‐SEM)

FE‐SEM (HITACHI S‐4160, Japan) was used to evaluate surface microstructure of red beetroot powder. After fixing the powder samples on aluminum plates, they were gold‐coated under a vacuum in a sputter (DSRI, Iran) to make them conductive. FE‐SEM microscopic images were obtained at 20‐kV accelerating voltage with magnification of ×600.

### 2.6 Statistical analyses

The experimental data were statistically analyzed in a completely randomized design in triplicate and presented as mean ± SD. Comparison of the means was conducted by one‐way analysis of variance (ANOVA) using SPSS 16.0 (SPSS, Inc., USA) followed by Duncan's multiple range test at *p* < .05.

## 3 MATHEMATICAL MODEL DEVELOPMENT

**FIGURE 2:**
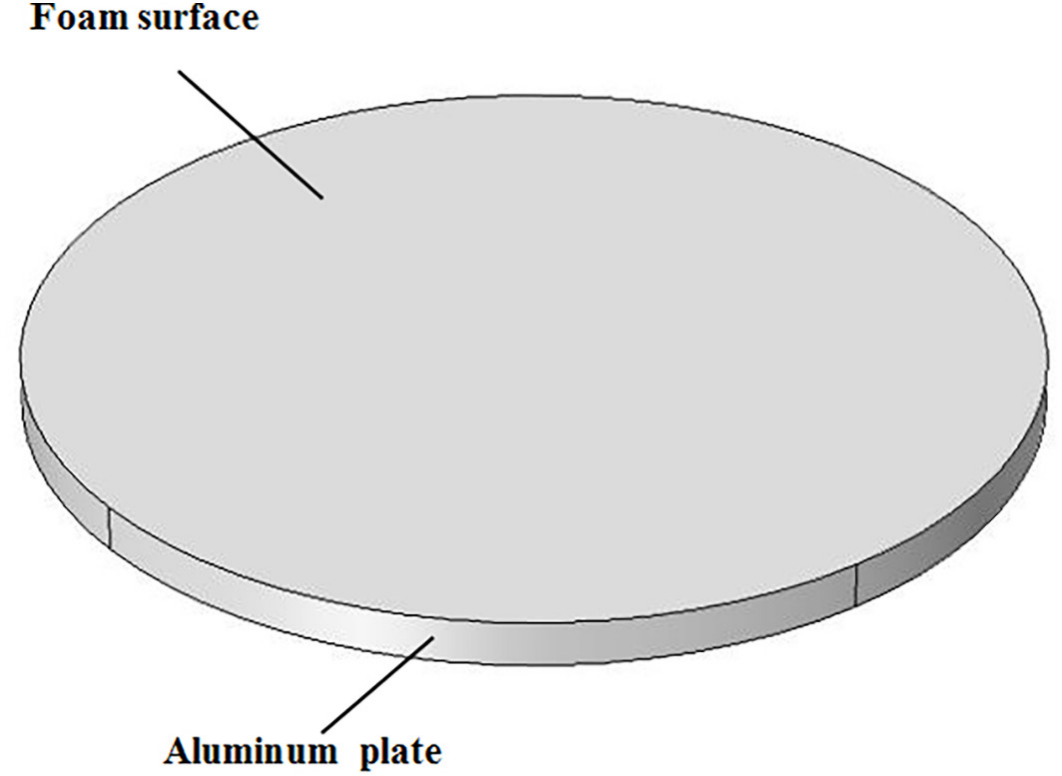
3D computational domain used for simulation.

Due to using round plates during foam‐mat drying, a 3D finite cylinder was considered as model geometry (Figure 2). However, since the height (thickness) of the foam (0.005 m) inside the cylinder was negligible compared to its diameter (0.15 m), the cylinder was assumed as infinite slab for calculation of the *D*_eff_ (Section 2.4.3). To develop the model, the following assumptions were made:

- Convective velocity within the foam was zero.
- Mass and heat generation and consumption within the foam were zero.
- Thermophysical properties of the foams were varied as a function of drying time.
- Initial distribution of moisture and temperature within the foam were homogeneous.
- Heat transfer within the viscous foam took place via conduction.
- Mass transfer within the viscous foam took place via diffusion.

### 3.1 Governing equations

The following equations for coupled transfer of mass and heat inside the foam were considered.

#### 3.1.1 Heat transfer

Fourier's law of heat conduction was used to obtain temperature distribution inside the foams (Jomlapelatikul et al., 2016; Ju et al., 2016; Khan et al., 2017):

(21) $$\rho_{f}c_{p.f}\left(\frac{\partial T_{f}}{\partial t}\right)=\nabla .\left(k_{f}\nabla T_{f}\right)$$

where *ρ* (kg/m^3^), *c*_*p.f*_ (J/kg K), *k*_*f*_ (W/m K), and *T*_*f*_ (°C) are the density, specific heat, thermal conductivity, and temperature of the foam at time *t*, respectively. The physical concept of this equation implies that heat inside the foam is transferred by conduction mechanism.

##### Determination of thermophysical properties

Thermophysical properties of foods are changed over drying and are influenced by temperature, chemical composition, and moisture content (Murakami & Okos, 1989). In this study, density (*ρ*), specific heat (*c*_*p*_), and thermal conductivity (*k*_*f*_) were determined using Choi and Okos (1986)‘s models based on the foam's chemical composition and temperature (Choi & Okos, 1986; Lemus‐Mondaca et al., 2013):

(22) $$\rho =\frac{\left(1-\varepsilon \right)}{\sum_{i=1}^{n}\frac{X_{i}}{\rho_{i}}}$$

(23) $$c_{P}=\sum_{i=1}^{n}C_{\mathrm{pi}}X_{i}$$

(24) Kf=∑i=1nKiXiρi∑i=1nXiρi

where *ε* is the porosity or air mass fraction of foam and *X*_*i*_ is the mass fraction of each component reported in Nistor et al. (2017) (Table 2).

**TABLE 2:** Proximate nutritional composition of raw red beetroot.

Component	Value (g/100 g)
Water	86.26 ± 1.56
Protein	1.59 ± 0.28
Fat	0.15 ± 0.04
Carbohydrate	9.42 ± 0.14
Fiber	2.52 ± 0.38
Ash	1.00 ± 0.10

#### 3.1.2 Mass transfer

Fick's second law of diffusion was used to obtain moisture distribution inside the foams (Jomlapelatikul et al., 2016; Ju et al., 2016; Khan et al., 2017):

(25) $$\frac{\partial C}{\partial t}+\nabla .\left(-D_{\mathrm{eff}}\nabla C\right)=0$$

where *C* (mol/m^3^) is moisture concentration, *t* (*s*) is time, and *D*_eff_ (m^2^/s) is the effective coefficient of moisture diffusivity. Equation 25 implies that moisture within the foam is transferred through diffusion during drying.

### 3.2 Initial and boundary conditions

The initial input parameters are listed in Table 3.

**TABLE 3:** Input parameters utilized for coupled heat and mass transfer modeling.

Parameter	Amount	Unit	Source
Initial moisture content	3.5657	kg/kg	Current study
Latent heat of evaporation	2,358,600	J/kg	Khan et al. (2017)
Molecular weight of water	0.018	kg/mol	Dehghannya et al. (2019)
Specific heat of air	1005.04	J/kg K	Onwude et al. (2018)
Velocity of drying air	1	m/s	Current study
Air density	1.073	kg/m^3^	Onwude et al. (2018)
Thermal conductivity of air	0.0287	W/m/K	Khan et al. (2017)
Initial foam temperature	20	°C	Current study
Foam thickness	0.005	m	Current study
Equilibrium moisture content	0.01	kg/kg	Current study
Dynamic viscosity of air	19.907 × 10^−6^	Pa. s	Dehghannya et al. (2019)
Initial density of foam	0.13778	kg/m^3^	Current study
Porosity of foam	0.866624	Dimensionless	Current study

#### 3.2.1 Initial conditions

The initial conditions were considered as follows:

(26) $$T_{f}=T_{0}$$

where *T*_*f*_ (°C) denotes foam temperature and *T*_0_ (°C) is initial ambient temperature.

(27) $$C=C_{0}$$

where *C* (mol/m^3^) is the moisture concentration and *C*_0_ (mol/m^3^) is the initial concentration of foam moisture.

#### 3.2.2 Boundary conditions

##### Heat transfer

Boundary condition at the foam surface (the only evaporating surface) was represented by (Jomlapelatikul et al., 2016; Ju et al., 2016; Khan et al., 2017; Onwude et al., 2018):

(28) $$n.\left(k_{f}\nabla T_{f}\right)=h\left(T_{\infty }-T_{f}\right)-h_{m}.\varphi .\rho_{\infty }\times \left(C-C_{e}\right)$$

where *n* is the boundary normal vector, *h* (W/m^2^ K) is the convective coefficient of heat transfer, *h*_*m*_ (m/s) is the convective coefficient of mass transfer, *T*_*∞*_ (°C) is the drying temperature, *φ* (J/kg) is the latent heat of vaporization, *ρ*_*∞*_ (kg/m^3^) is the air density, *C*_*e*_ (mol/m^3^) is the equilibrium moisture concentration, and *C* (mol/m^3^) is the foam moisture concentration. The first and second terms on the right‐hand side indicate convective heating and evaporative cooling, respectively. This boundary condition suggests that total flux of heat on the surface of foam corresponds to the difference between inward flux of heat due to convection (from air to foam surface) and outward heat flux because of vaporization (from foam surface to air).

The boundary condition at the sides and bottom of the plates was considered as follows:

(29) $$T_{f}=T_{\infty }$$

where *T*_*∞*_ (°C) is the temperature of air and *T*_*f*_ (°C) is the temperature of foam.

### 3.3 Mass transfer

The boundary condition on the foam surface was specified as (Ju et al., 2016; Khan et al., 2017; Onwude et al., 2018):

(30) $$n.\left(D_{\mathrm{eff}}\nabla C\right)=h_{m}\left(C_{\infty }-C\right)$$

where *n* is the boundary normal vector, *h*_*m*_ (m/s) is the convective coefficient of mass transfer, and *C*_*∞*_ (mol/m^3^) is the ambient air moisture concentration. This boundary condition indicates that mass transfer on the foam surface is done through convection to the surrounding air.

The boundary condition at the sides and bottom of the plates was considered as:

(31) $$\frac{\partial C}{\partial t}=0$$

### 3.4 Coefficients of mass and heat transfer

The convective coefficients of mass and heat transfer, *h*_*m*_ and *h*, respectively, were calculated through dimensionless Nusselt (Nu), Reynolds (Re), Sherwood (Sh), and Schmidt (Sc) numbers (Franco et al., 2015; Khan et al., 2017):

(32) Nu=0.664Re1/2Pr1/3

(33) Re=ρ∞u∞dμ∞

(34) Sh=0.664Re1/2Sc1/3

(35) $$\mathrm{Sc}=\frac{\mu_{\infty }}{\rho_{\infty }\times D_{\mathrm{AB}}}$$

(36) $$h_{m}=\frac{\mathrm{sh}\times D_{\mathrm{AB}}}{d}$$

(37) h=hm×ρ∞×cP,∞×αDAB23

where *ρ*_*∞*_ (kg/m^3^) is the air density, *u*_*∞*_ (m/s) is the air velocity, *d* (mm) is the foam thickness, *μ*_*∞*_ (Pa s) is the dynamic viscosity of air, *D*_AB_ (m^2^/s) is the coefficient of vapor diffusivity in air, and *α* (m^2^/s) is the thermal diffusivity.

*D*_AB_ and *α* were calculated by Dehghannya et al. (2019):

(38) $$D_{\mathrm{AB}}=\frac{2.26}{P}{\left(\frac{T+273.18}{273.18}\right)}^{1.81}$$

where *T* (°C) is the air temperature and *P* (Pa) is the pressure.

(39) $$\alpha =\left(\frac{k_{\infty }}{\rho_{\infty }c_{P,\infty }}\right)$$

where *ρ*_*∞*_ (kg/m^3^) is the air density, *c*_*p∞*_ (J/kg K) is the air‐specific heat, and *k*_*∞*_ (W/m K) is the air thermal conductivity.

### 3.5 Numerical simulation

An unsteady‐state coupled mass and heat transfer mathematical model during foam‐mat drying of red beetroot was developed and solved using COMSOL Multiphysics Software (version 5.2a). Elements with triangular meshes and time steps of 30 s were used to predict moisture and temperature profiles. To obtain mesh‐independent solutions, various mesh sensitivity tests were performed at various temperatures (50, 60, and 70°C). Details of complete mesh statistical results are given in Table 4. The simulations were done on a 64‐bit computer with intel® Core™ i7‐6700HQ/BGA CPU with 3.5 GHz processor speed and 8.00 GB installed RAM. Various simulation steps were as follows:

1. Building model geometry.
2. Defining input parameters.
3. Meshing.
4. Defining thermophysical properties.
5. Defining boundary conditions.
6. Predicting initial temperature and moisture distributions.
7. Updating input parameters and variables (thermal properties).
8. Obtaining final temperature and moisture profiles.
9. Validating model experimentally.

**TABLE 4:** Mesh sensitivity analysis for heat and mass transfer modeling.

Mesh statistics	Mesh type		
	Normal	Fine	Finer
Number of domain elements	1443	2264	3279
Number of boundary elements	1000	1556	2218
Number of edge elements	100	116	140
Minimum element quality	0.06505	0.1587	0.1696
Degrees of freedom	3493	5429	7792
Time length of simulation	16 s	21 s	31 s

The mass transfer model validation was done explicitly via moisture content measurements (Dehghannya et al., 2019). Since the temperature of foams reached a constant value (air temperature) at the very first moments of drying due to a low foam thickness (5 mm), the heat transfer model validation was performed indirectly by employing the experimental model validation of mass transfer. Therefore, empirical measuring of foam temperature was not carried out.

## 4 RESULTS AND DISCUSSION

### 4.1 Drying kinetics of foam

**FIGURE 3:**
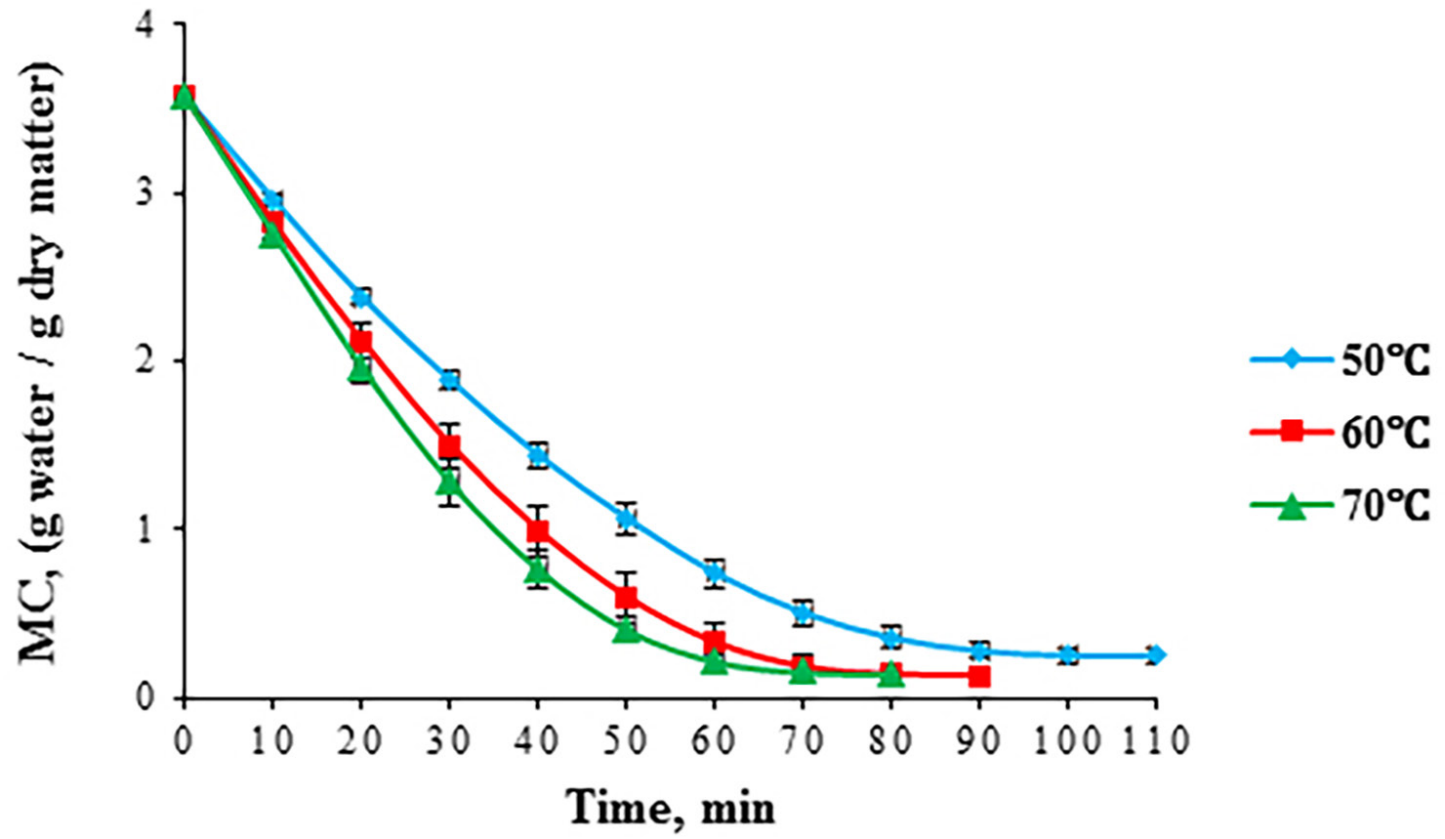
Moisture content of foam‐mat‐dried red beetroot pulp vs. drying time at various drying temperatures.

**FIGURE 4:**
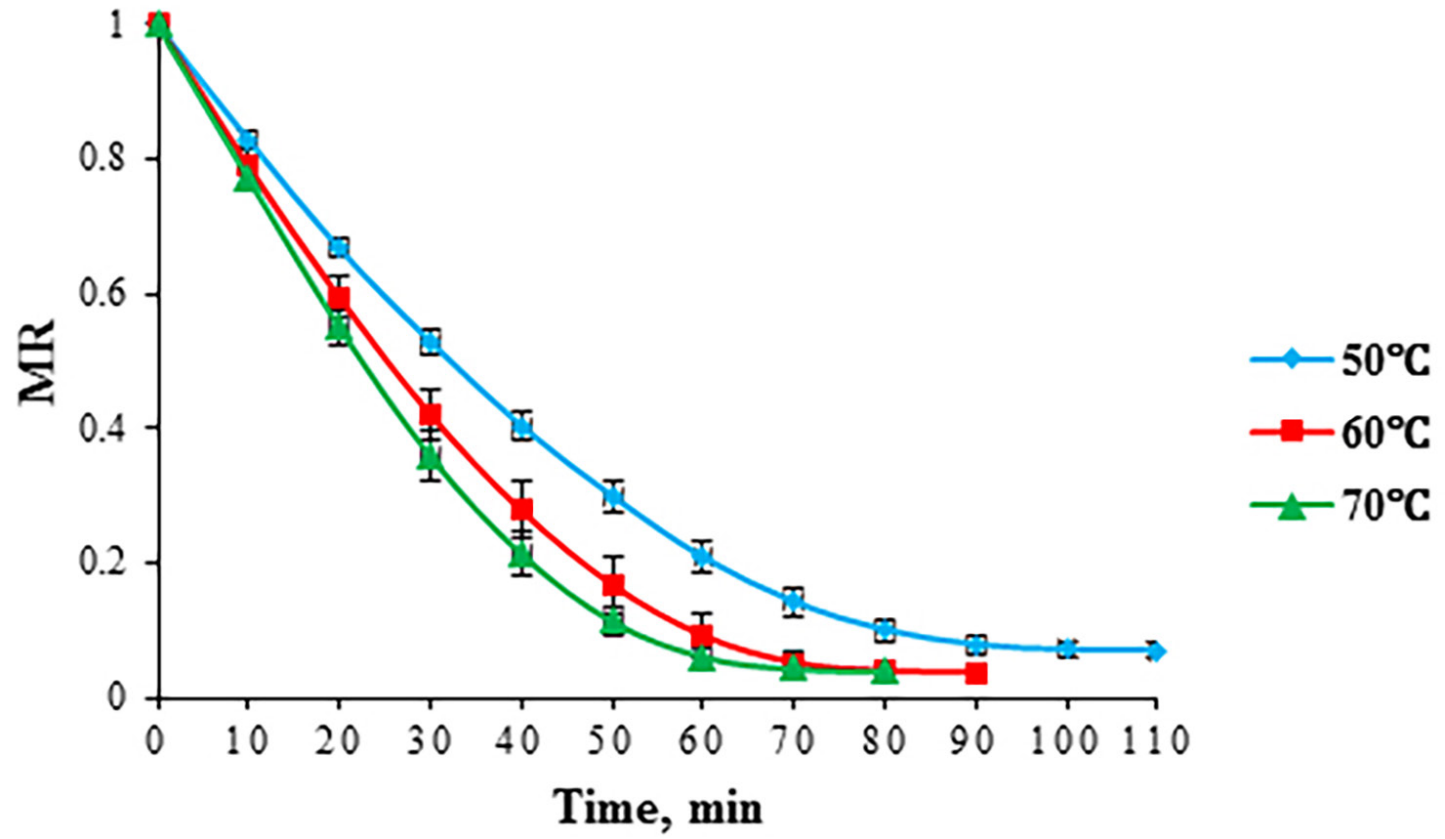
Moisture ratio of foam‐mat‐dried red beetroot pulp vs. drying time at various drying temperatures.

The changes in the moisture content (MC) of foams during drying at various temperatures (50, 60, and 70°C) are indicated in Figure 3. An increase in temperature gave rise to a reduction in drying time. Average values of the MC at various drying temperatures are also listed in Table 5. By increasing temperature from 50 to 70°C, the mean value of MC dropped by about 8% (Table 5). Similarly, the moisture ratio (MR) of foams gradually decreased with drying time until the equilibrium moisture content was reached (Figure 4). Changes in drying temperature exerted an insignificant impact (*p* > .05) on the mean values of MR (Table 5).

**TABLE 5:** Mean values of moisture content (MC), moisture ratio (MR), and drying rate (DR) for red beetroot foams at various drying temperatures.

Temperature (°C)	MC (g water/g dry matter)	MR (dimensionless)	DR (g water/g dry matter)/min
50	1.294^a^ ± 0.016	0.363^a^ ± 0.004	0.029^a^ ± 0.002
60	1.235^a^ ± 0.082	0.346^a^ ± 0.023	0.034^b^ ± 0.000
70	1.184^a^ ± 0.059	0.331^a^ ± 0.017	0.037^c^ ± 0.002

*Note*: Different superscript letters in the same columns denote a significant difference (*p* < .05).

**FIGURE 5:**
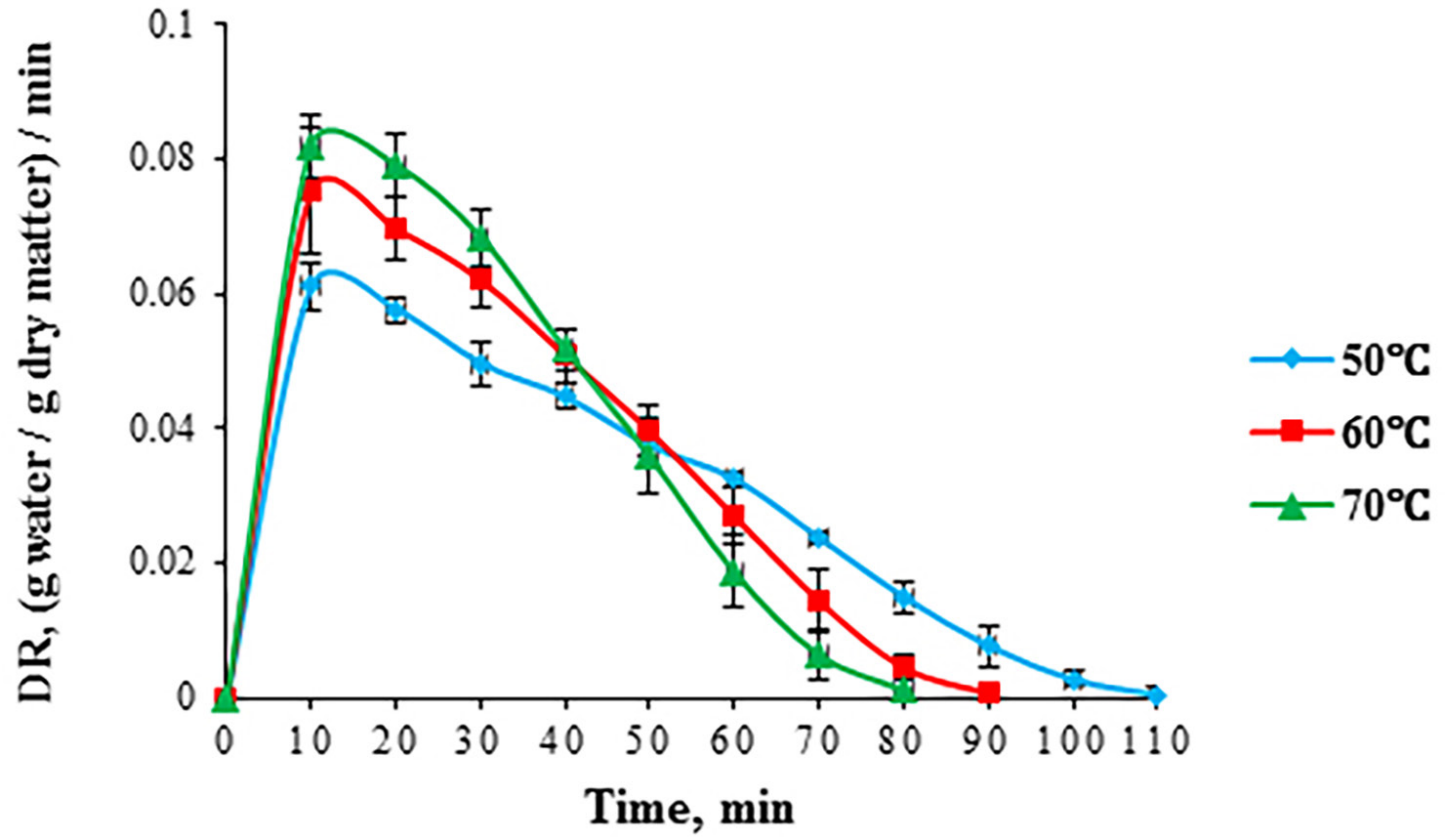
Drying rate of foam‐mat‐dried red beetroot pulp vs. drying time at various drying temperatures.

By increasing drying temperature, drying rate (DR) increased and the required drying duration was shortened (Figure 5). Two distinguished stages were observed during drying. In the early stage of drying (within the first minute), the DR was higher than in the later stages regarding all three temperatures. This result was attributed to a greater unbound water on the foam surface; thereby, a higher moisture concentration difference between air and foam, leading to a low internal resistance for moisture transfer. During this stage, the foam remains at the wet‐bulb temperature thanks to the evaporative cooling for a short time. In the second stage, the DR after attaining the highest point, gradually decreased due to the lack of unbound water on the foam surface, resulting in a high internal resistance for moisture transfer (Dehghannya et al., 2019). Results showed that drying operation occurred in the falling‐rate period and the constant‐rate period was not observed considering different temperatures (Figure 5). During the falling‐rate period, diffusion is the main physical flow mechanism controlling internal moisture movement to the surface (Chaux‐Gutiérrez, Santos, et al., 2017; Franco et al., 2017). In this period, the surface of the foam is almost dry, and the foam's temperature rises toward the dryer temperature due to the lack of evaporative cooling. By increasing drying temperature from 50 to 70°C, DR significantly increased by 27.58% (Table 5). This outcome was in agreement with many reported findings in the literature for other food materials where higher temperature increases thermal difference between drying air and foam, which can lead to higher drying rates (Demiray et al., 2017; Franco et al., 2015; Ju et al., 2016).

**FIGURE 6:**
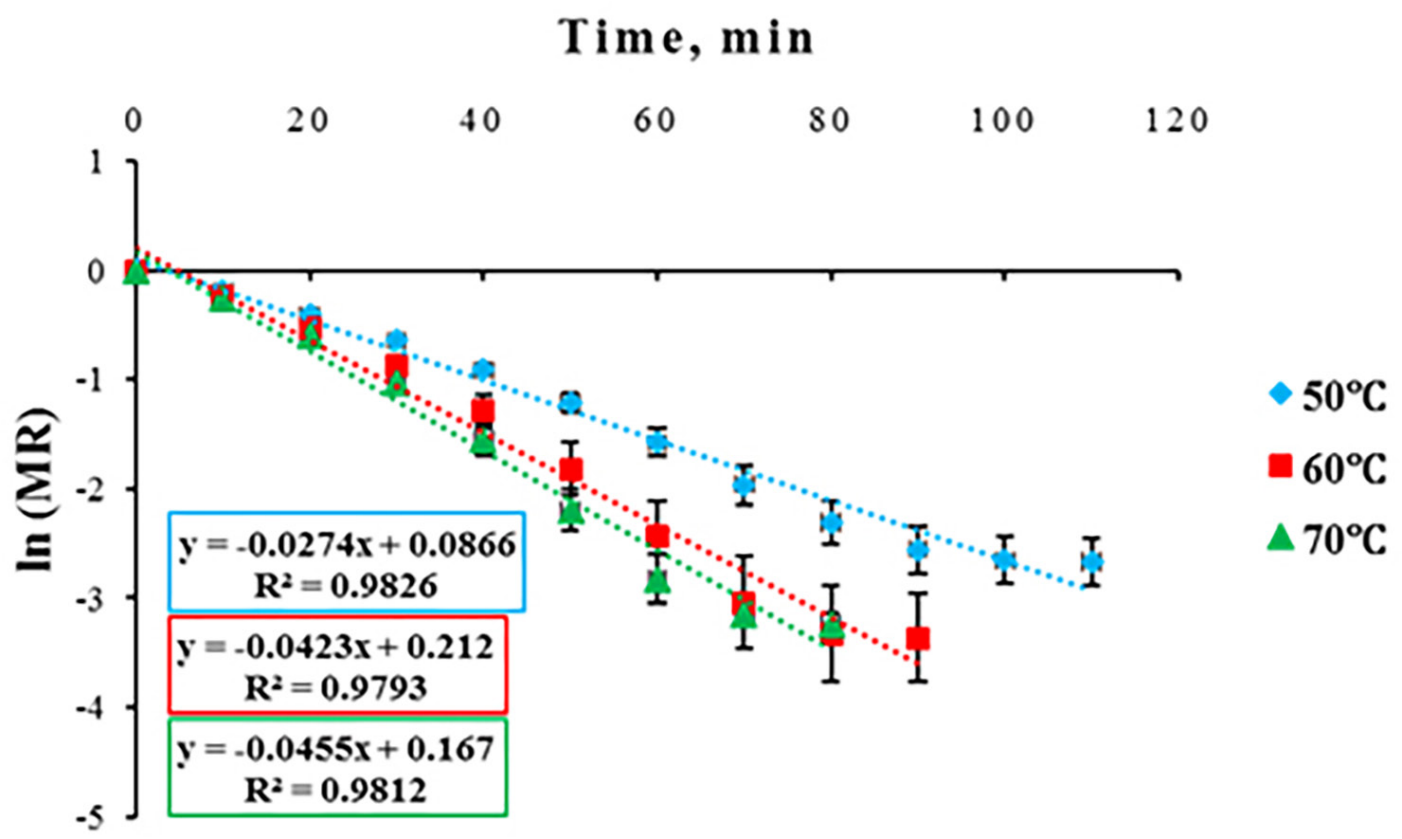
Variations in ln (MR) vs. drying time at different drying temperatures.

Effective moisture diffusivity coefficient (*D*_eff_) is a key transfer attribute explaining a conductive term responsible for all moisture transfer mechanisms including capillary flow, molecular diffusion, and hydrodynamic transfers from food products during drying (Asokapandian et al., 2016; Castro et al., 2018). Drying rate is influenced by *D*_eff_ (Khan et al., 2017; Kumar et al., 2015). Results showed that ln (MR) values dropped with a sharper slope at higher drying temperatures (Figure 6). *D*_eff_ changes during drying on account of the influences of initial MC of the material, process temperature, density, porosity, and interaction between substance constituents such as protein, fat, and starch with water (Franco et al., 2017). Results showed that by increasing drying temperature from 50 to 70°C, *D*_eff_ significantly increased (Table 6). This could be linked to an increase in the water vapor pressure gradient between the inner and surface sections of the foams at higher temperatures, leading to the enhancement of *D*_eff_ (Dehghannya et al., 2019). In general, values of moisture diffusivity for different foodstuffs vary between 10^−11^ and 10^−9^ m^2^/s (Asokapandian et al., 2016).

**TABLE 6:** Effective moisture diffusivity coefficients (*D*_eff_) at various drying temperature and activation energy (*E*_*a*_) for red beetroot foams.

Temperature (°C)	*D*_eff_ (m^2^/s)	*E*_*a*_ (kJ/Mol)
50	5.218 × 10^−9a^ ± 5.298 × 10^−10^	23.538
60	7.150 × 10^−9ab^ ± 9.989 × 10^−10^	
70	8.689 × 10^−9b^ ± 1.780 × 10^−9^	

*Note*: Different superscript letters in the same columns denote a significant difference (*p* < .05).

**FIGURE 7:**
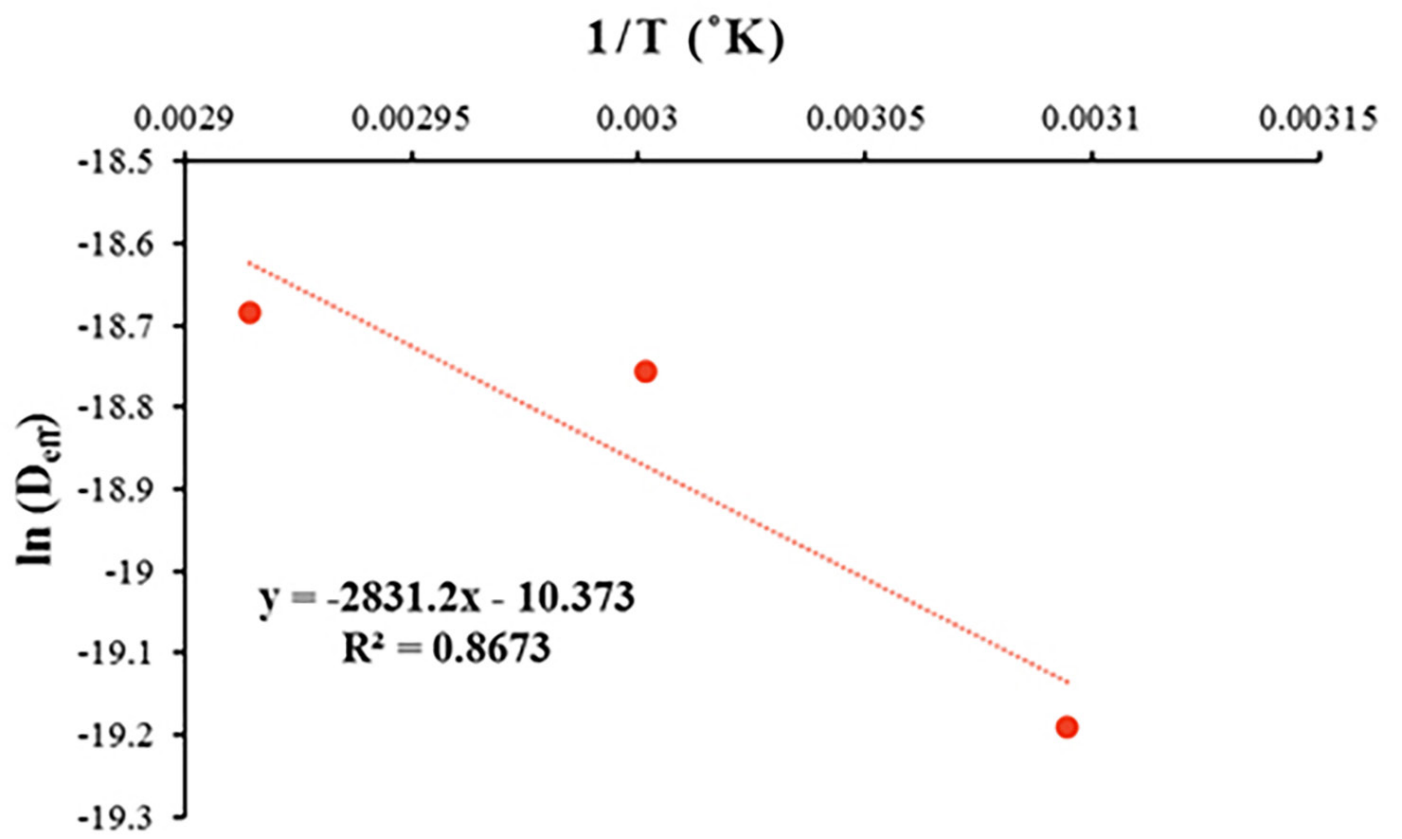
Arrhenius‐type relationship between effective moisture diffusivity coefficient (*D*_eff_) and absolute temperature inverse.

Activation energy (*E*_*a*_) is an index of the energy necessary to commence internal moisture transfer of food material to its surface. The general range of *E*_*a*_ for different food materials and agriculture products is from 12.7 to 110 kJ/mol (Aral & Beşe, 2016; Horuz et al., 2017; Olanipekun et al., 2015). The *E*_*a*_ was determined using the Arrhenius relationship via plotting ln *D*_eff_ vs. 1/(*T* + 273.15) (Figure 7). The value of *E*_*a*_ was 23.538 kJ/mol (Table 6).

### 4.2 Functional properties of powder

#### 4.2.1 Bulk and tapped density

Bulk density (*ρ*_*B*_) is an essential indicator for powders due to its effect on packaging, storage, transportation, and reconstitution of powders (Seerangurayar et al., 2017). Increased density after mechanical tapping or gentle shaking is expressed as tapped density (*ρ*_*T*_). The tapped density is a major criterion to evaluate the packaging behavior of the powders, even though other essential factors, including surface roughness or morphology, particle shape, size and distribution, cohesiveness, and surface charges affect this behavior (Bhandari, 2013). Results showed that the *ρ*_*B*_ and *ρ*_*T*_ of powders were significantly declined by increasing drying temperature (Table 7). Santhalakshmy et al. (2015) reported the same trend for spray‐dried Jamun powder, where a lower temperature resulted in a higher tapped density. Similar observations were also stated by Tze et al. (2012) for spray‐dried pitaya. Chegini and Ghobadian (2005) observed that a higher drying temperature lessened the moisture content of powder, which caused the bulk density to reduce since water is noticeably denser compared to dry solids of foods. Similarly, Asokapandian et al. (2016) stated that with increasing drying temperature, bulk density of muskmelon powder declined due to higher moisture evaporation rates at higher temperatures.

**TABLE 7:** Impact of drying temperature on qualitative characteristics of red beetroot powder.

Property	Temperature (°C)		
	50	60	70
*ρ*_*B*_ (g/cm^3^)	0.549^b^ ± 0.003	0.560^c^ ± 0.003	0.541^a^ ± 0.003
*ρ*_*T*_ (g/cm^3^)	0.623^b^ ± 0.004	0.627^b^ ± 0.002	0.603^a^ ± 0.002
*ρ*_*p*_ (g/cm^3^)	1.363^a^ ± 0.011	1.362^a^ ± 0.006	1.361^a^ ± 0.020
*ε* (dimensionless)	0.596^ab^ ± 0.005	0.589^a^ ± 0.003	0.603^b^ ± 0.004
CI (%)	11.917^b^ ± 0.454	10.739^a^ ± 0.370	10.386^a^ ± 0.250
HR (dimensionless)	1.135^b^ ± 0.006	1.120^a^ ± 0.005	1.116^a^ ± 0.003
WAI (dimensionless)	3.204^a^ ± 0.260	3.048^a^ ± 0.188	2.999^a^ ± 0.151
WSI (%)	75.533^a^ ± 1.007	76.833^a^ ± 0.961	76.333^a^ ± 0.757
MC (%, wb)	6.308^b^ ± 0.097	6.200^ab^ ± 0.098	6.092^a^ ± 0.046

*Note*: Different superscript letters in the same rows denote a significant difference (*p* < .05).

#### 4.2.2 Particle density

Particle density is related to actual solid density and does not regard interparticle spaces (Santana et al., 2013; Seerangurayar et al., 2017; Tonon et al., 2010). Particle density showed a decreasing behavior with increasing drying temperature (Table 7). In a similar study, Dehghannya et al. (2019) showed that changes in temperature from 50 to 70°C gave rise to significant reductions in particle density of foam‐mat‐dried lime juice powder as a consequence of higher moisture evaporation rates at higher drying temperatures.

#### 4.2.3 Bulk porosity

Porosity is a main physical characteristic affecting stability of food powders during storage. Powders with higher porosity have higher air spaces between particles, and thus, higher oxygen available for degradation reactions (Franco et al., 2016; Santhalakshmy et al., 2015; Zotarelli et al., 2017). According to Table 7, as drying temperature increased, bulk porosity initially decreased and then started to increase. Dehghannya et al. (2019) observed that raising drying temperature induced a significant decline in bulk porosity of foam‐mat‐dried lime juice powder. Bulk porosity reversely corresponds to bulk density, suggesting that air being trapped in the foam causes air to accumulate within the dried particles, which makes them less close‐packed and more porous (Franco et al., 2016; Seerangurayar et al., 2017).

#### 4.2.4 Flowability

Flowability of powders is a key and complicated attribute determined by Carr index (CI) or compressibility index and Hausner ratio (HR) or cohesiveness index. It depends on surface composition of a powder; for instance, fiber, fat, protein, and moisture as well as particle shape, size, size distribution, surface structure, particularly surface roughness and angle of repose (Aziz et al., 2018; Bhandari, 2013; Saifullah et al., 2016; Seerangurayar et al., 2017). As the drying temperature increased, both CI and HR decreased (Table 7). A rise in the temperature from 50 to 70°C significantly lowered CI and HR by 12.85 and 1.67%, respectively, while increasing temperature from 60 to 70°C had no significant effect on both the CI and HR. In general, the powders obtained at 70°C had good flowability properties compared to the ones dried at 50 and 60°C. The same behaviors were observed by Asokapandian et al. (2016) and Dehghannya et al. (2019) for foam‐mat‐dried muskmelon and lime juice powders, respectively. Moreira et al. (2009) showed that elevating drying temperature lessened the moisture content of powder, leading to the enhancement of the flowability characteristic.

#### 4.2.5 Water absorption index (WAI) and water solubility index (WSI, %)

Water absorption is a fundamental index of powders capability to absorb water, and is directly linked with their hydration capacity (Franco et al., 2016; Shaari et al., 2018). Increasing drying temperature caused a decline in WAI (Table 7). Similarly, Asokapandian et al. (2016), Dehghannya et al. (2019), and Franco et al. (2016) observed a decreasing trend in WAI with an increase in drying temperature. Solubility of powders relies on composition, particle size, moisture content, and physical state of the particles. For example, powders composed of carbohydrates such as maltodextrin and gum Arabic possess a high solubility. On the contrary, the powders comprising proteins, for instance, gelatin and soy protein isolate retain a low solubility (Tontul & Topuz, 2017). Results showed that WSI first increased as drying temperature went up from 50 to 60°C, and then diminished when temperature increased from 60 to 70°C. An increase in temperature intensifies protein denaturation, which results in a decrease in WSI (Saifullah et al., 2016). Similar findings were observed by Abbasi and Azizpour (2016) who investigated foam‐mat drying of sour cherry and reported that with an increase in temperature from 50 to 65°C, WSI increased, and then, with raising temperature from 65 to 80°C, it began to drop. Salahi et al. (2017), Chaux‐Gutiérrez, Pérez‐Monterroza, et al. (2017); Chaux‐Gutiérrez, Santos, et al. (2017), and Franco et al. (2016) reported similar results for foam‐mat‐dried cantaloupe, mango, and yacon juice, respectively.

#### 4.2.6 Moisture content (MC)

Moisture is one of the essential factors influencing powder stickiness, flowability, and storage stability on account of its impacts on glass transition temperature (*T*_*g*_) and crystallization behavior (Aziz et al., 2018). Table 7 shows that increasing drying temperature resulted in a lower MC of the powders. Similar results were reported by Salahi et al. (2017) for foam‐mat‐dried cantaloupe powder. A higher drying temperature enhances heat transfer to the sample, which amplifies moisture migration inside the foam, accelerating surface moisture vaporization. This gave rise to the production of powders with lower moisture content (Salahi et al., 2017; Tontul & Topuz, 2017).

#### 4.2.7 Color

Color is a main quality parameter in foodstuffs playing a significant role in product selection and acceptance by customers (Deng et al., 2019; Goñi & Salvadori, 2017). Basically, color represents the quality, freshness, and safety of foods, which greatly affects their desirability and price (Selig et al., 2018; Yang et al., 2018). With enhancing drying temperature from 50 to 70°C, lightness (L*) significantly increased (Table 8). L* in foods not only depends on the concentration and type of pigments but also on the concentration of surface moisture (Franco et al., 2016). Besides, as the temperature of drying air increased, a descending trend in redness (a*) was observed (Table 8). Bazaria and Kumar (2016) and Kowalski and Szadzińska (2014) noticed similar results for beetroot powders, attributing the observations to decomposition of betalains at higher drying temperatures. Nistor et al. (2017) observed that red beetroots dried at 60°C had higher betacyanins (red) and betaxanthins (yellow) than those dried at 70°C. Moreover, increasing drying temperature decreased yellowness (b*) (Table 8). Increasing drying temperature from 50 to 60°C gave rise to a significant reduction in the yellowness of powders by 23.15%. (Table 8). Physical, microbial, and chemical variations, which take place in the course of growth, maturation, postharvest handling, and processing, play crucial roles in the color development of food powders (Pathare et al., 2013). Of other factors influencing the color of powders are drying type and parameters such as velocity and temperature of hot air (Dehghannya et al., 2019).

**TABLE 8:** Color parameters of red beetroot powder including lightness (L*), redness (a*), and yellowness (b*) as influenced by drying temperature.

Temperature (°C)	CIELAB color coordinates		
	L*	a*	b*
50	36.306^a^ ± 0.668	32.515^a^ ± 0.840	5.567^b^ ± 0.404
60	38.929^b^ ± 2.008	31.559^a^ ± 2.909	4.690^ab^ ± 0.757
70	38.967^b^ ± 0.498	30.233^a^ ± 0.751	4.278^a^ ± 0.481

*Note*: Different superscript letters in the same columns denote a significant difference (*p* < .05).

The total color difference (Δ*E*) is a colorimetric parameter mostly utilized to determine alterations of color in foodstuffs upon processing. A higher Δ*E* implies higher variations in color from the reference material (Shewale & Hebbar, 2017). Chroma or saturation index (C*) is a quantitative characteristic of color which indicates the degree of intensity or purity of color (Chaux‐Gutiérrez, Santos, et al., 2017). Hue angle (*H*°) is a color qualitative attribute (Pathare et al., 2013) and browning index (BI) shows brown color purity, a key indicator of browning in food products containing sugar (Caliskan & Dirim, 2016). The impacts of different drying temperatures on color indexes (Δ*E*, C*, *H*°, and BI) of foam‐mat‐dried red beetroot powder are listed in Table 9. As the drying temperature went up from 50 to 60°C, ΔE increased but it started to decline by enhancing temperature from 60 to 70°C. C*, *H*°, and BI are reduced by increasing temperature. With increasing temperature from 50 to 60°C, *H*° and BI significantly decreased by 19.58 and 16.35%, respectively. Higher temperatures result in enzymatic and nonenzymatic browning (Millard reactions) and lead to variations of color during drying of food materials (Franco et al., 2016). Similar to our results, Abbasi and Azizpour (2016) reported that an increase in temperature decreased the browning index of foam‐mat‐dried sour cherry powder.

**TABLE 9:** Color indices of red beetroot powder including total color difference (Δ*E*), Chroma index (C*), hue angle (*H* **°**), and browning index (BI) at various drying temperatures.

Temperature (°C)	Color parameters			
	Δ*E*	C*	*H*°	BI
50	34.848^a^ ± 0.855	32.989^a^ ± 0.883	9.513^b^ ± 0.182	73.660^b^ ± 2.322
60	35.722^a^ ± 1.039	31.914^a^ ± 2.881	9.063^b^ ± 0.556	65.087^ab^ ± 7.781
70	34.608^a^ ± 0.587	30.538^a^ ± 0.685	7.650^a^ ± 0.362	61.614^a^ ± 0.105

*Note*: Different superscript letters in the same columns denote a significant difference (*p* < .05).

#### 4.2.8 Microstructure and morphology of powder particles

**FIGURE 8:**
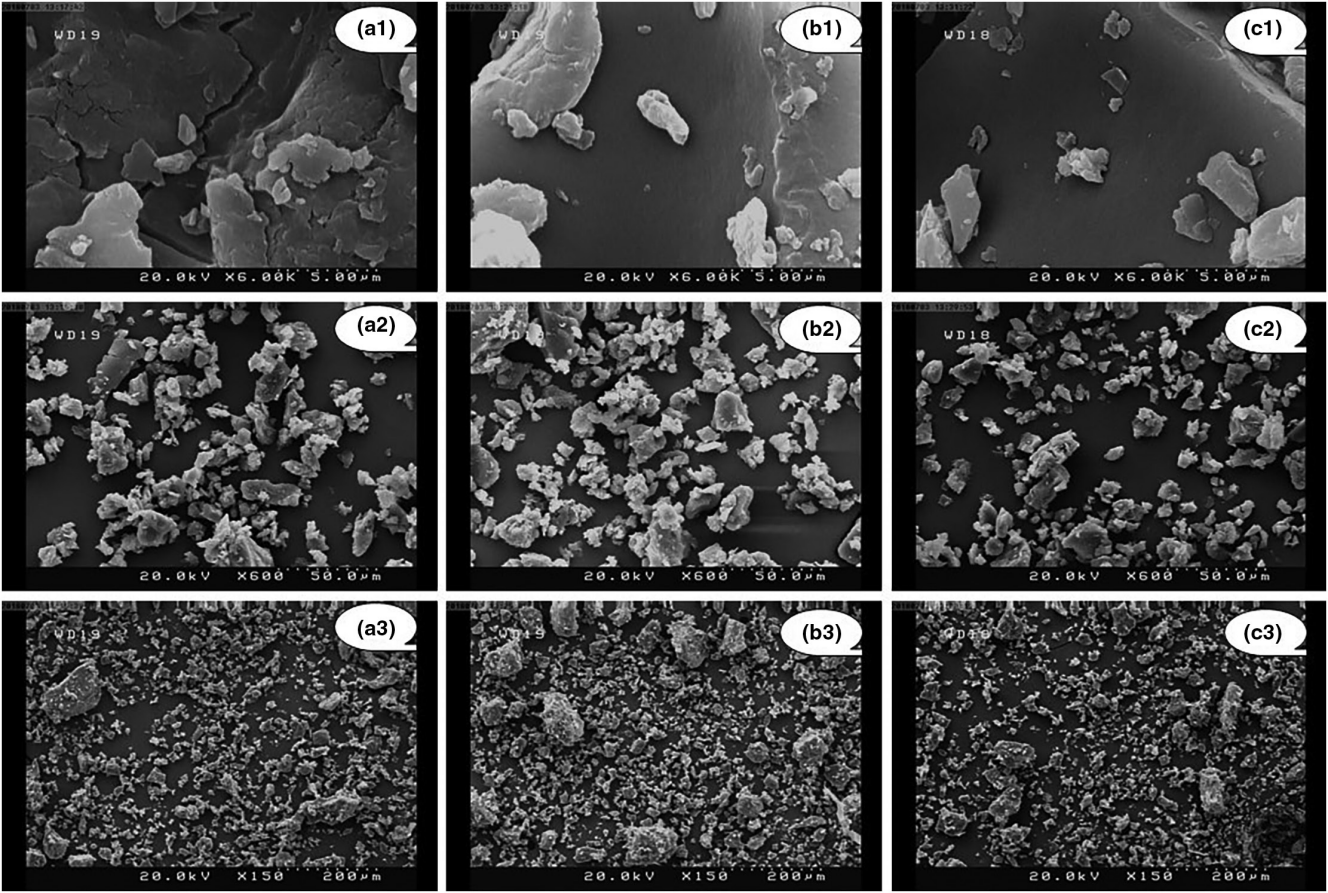
Field emission scanning electron microscope images of the foam‐mat‐dried red beetroot powder particles at scales of 5 μm (a_1_ to c_1_), 50 μm (a_2_ to c_2_), and 200 μm (a_3_ to c_3_) dried at (a) 50°C (a_1_ to a_3_); (b) 60°C (b_1_ to b_3_); and (c) 70°C (c_1_ to c_3_).

Particle size, shape, size distribution, surface characteristics, and microstructure are key properties in determining behavior of powder particles that can considerably influence further processing, ease of handling, packaging, stability, and fluidity. Various factors such as powder manufacturing processes, composition, and type affect the aforementioned properties (Aziz et al., 2018). Images obtained from field emission scanning electron microscopy (FE‐SEM) demonstrated that by increasing drying temperature from 50 to 70°C, wrinkling, cracking, and roughness of the powder particles decreased and particles with relatively fine and smooth surfaces were obtained, resulting in a higher flowability (Figure 8(a_1_ to c_1_)). This coincides with the results of Dantas et al. (2018), Jafari et al. (2017), and Tontul and Topuz (2017) who stated that at higher drying temperatures, smooth and hard crusts on the surface of particles were rapidly formed due to higher moisture evaporation rates, which prevented shrinkage of particles while drying. Azizpour et al. (2016) and Salahi et al. (2017) asserted that the porosity of foam‐mat‐dried structure increased by increasing drying temperature. The researchers ascribed this trend to a decline in processing time at higher drying temperatures, leading to a lower collapse of bubbles.

Additionally, as can be noticed in Figure 8 (micrographs: a_2_ to c_2_ and a_3_ to c_3_), all powder particles had irregular and fibrous structures with no homogeneity. The presence of larger particles could be linked to fibrous parts of raw red beetroot, which were hard to grind into small particles (Burgain et al., 2017). Similarly, Franco et al. (2016) reported that drying conditions including air temperature and foam thickness did not influence microstructural properties and porosity of yacon juice powder particles. They observed all samples with irregular shapes and lack of uniformity.

### 4.3 Simulated moisture and temperature profiles

**FIGURE 9:**
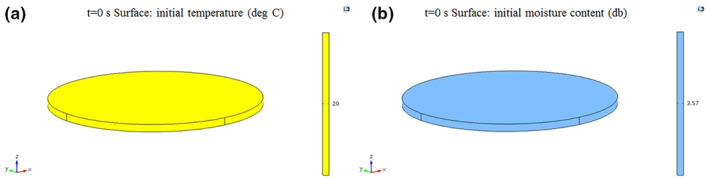
Temperature (a) and moisture (b) profiles at the initial stage of drying process.

**FIGURE 10:**
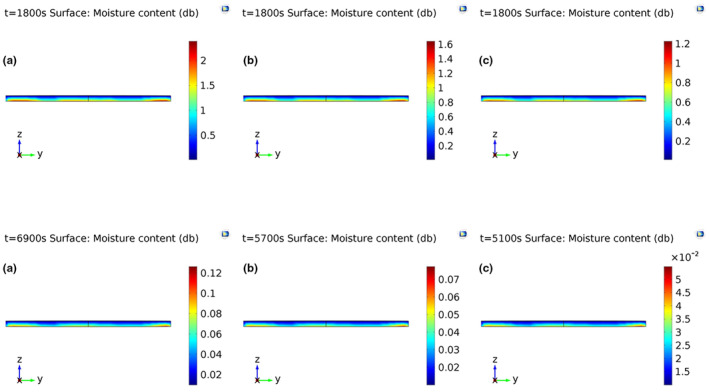
2D internal moisture profiles after 1800 s and after termination of drying (6900, 5700, and 5100 s) at drying temperatures of 50°C (a), 60°C (b), and 70°C (c), respectively.

**FIGURE 11:**
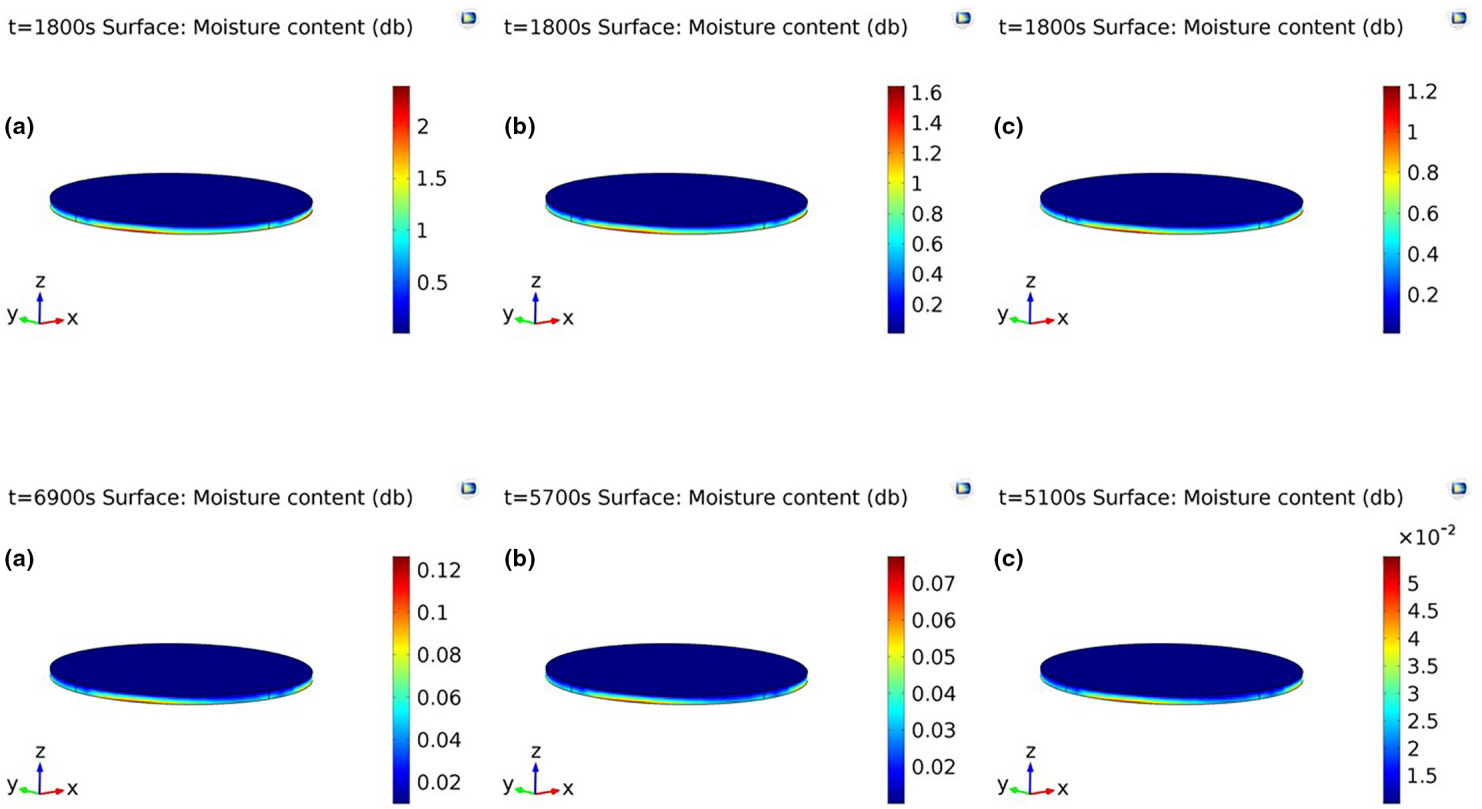
3D moisture profiles after 1800 s and after termination of drying (6900, 5700, and 5100 s) at drying temperatures of 50°C (a), 60°C (b), and 70°C (c), respectively.

**FIGURE 12:**
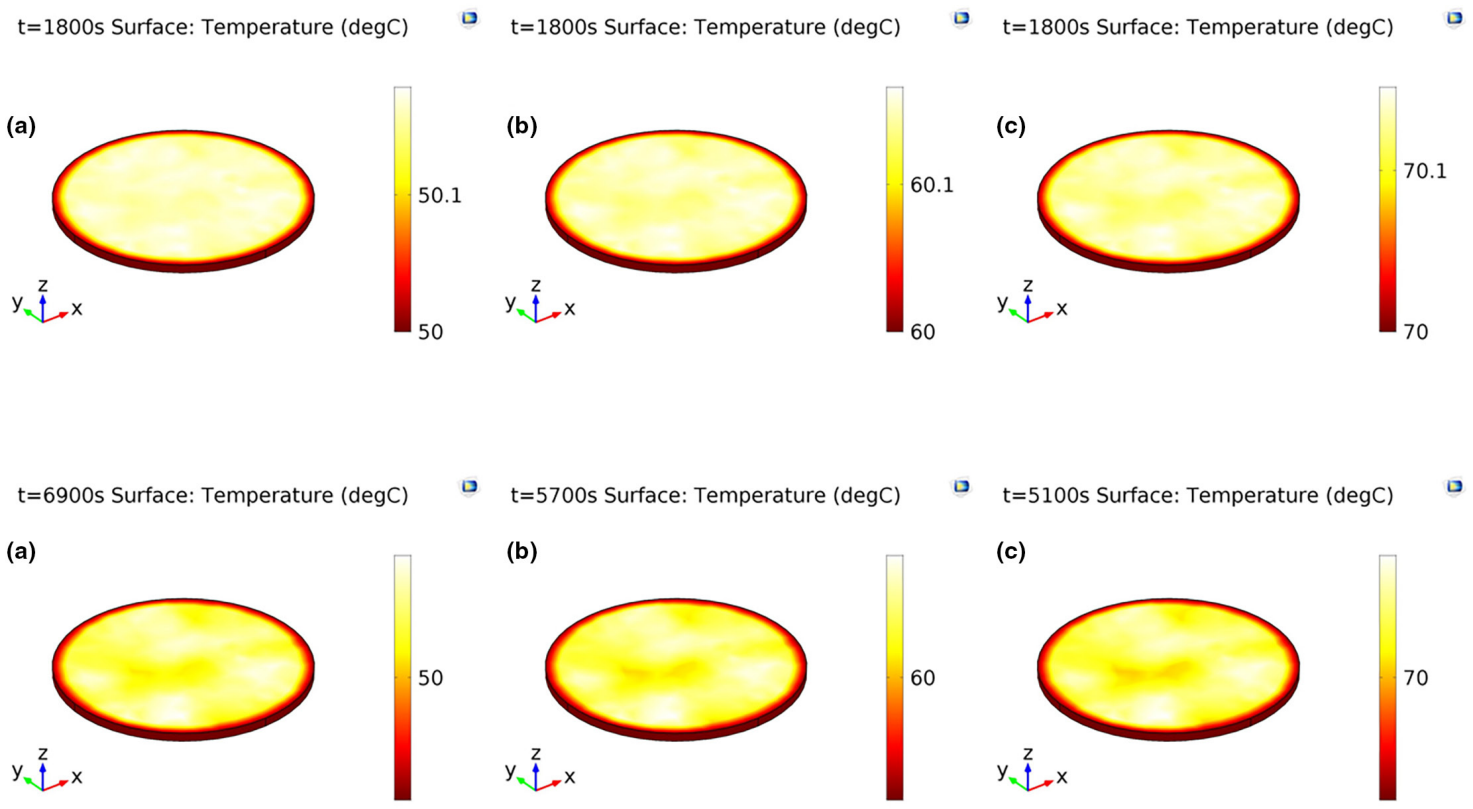
3D temperature profiles after 1800 s and after termination of drying (6900, 5700, and 5100 s) at drying temperatures of 50°C (a), 60°C (b), and 70°C (c), respectively.

Temperature and moisture distributions throughout the foam were almost uniform at the onset of drying (Figure 9). The initial moisture content (MC) and temperature of the foams were 3.57% (db) and 20°C, respectively. Two‐ and three‐dimensional internal moisture profiles after 1800 s and after termination of the drying process at different temperatures are depicted in Figures 10 and 11, respectively. According to these figures, moisture was transferred from the inner to the exterior part of the foams during drying. This was because the surface was the only air–foam interface in which evaporation occurred (Dehghannya et al., 2019). The drying time to reach the intended final moisture content at 50, 60, and 70°C was 115, 95, and 85 min, respectively. Moreover, as can be seen in Figures 10 and 11, moisture distribution of the foams dehydrated at 70°C was more uniform than those dehydrated at 50 and 60°C. This was because the moisture difference between the bottom and surface of the samples dried at 70°C was lower as compared to others. At the last moment of drying, the highest and lowest MC at the bottom and on the surface of the foams were 0.096 and 0.01 (db) at 50°C, 0.065 and 0.01 (db) at 60°C, and 0.047 and 0.01 (db) at 70°C, respectively. Figure 12 shows three‐dimensional temperature profiles after 1800 s and after termination of drying. The temperature difference between the upper and inner parts of the foams was negligible due to the small foam thickness (5 mm) used during drying (Figure 12).

### 4.4 Experimental model validation

**FIGURE 13:**
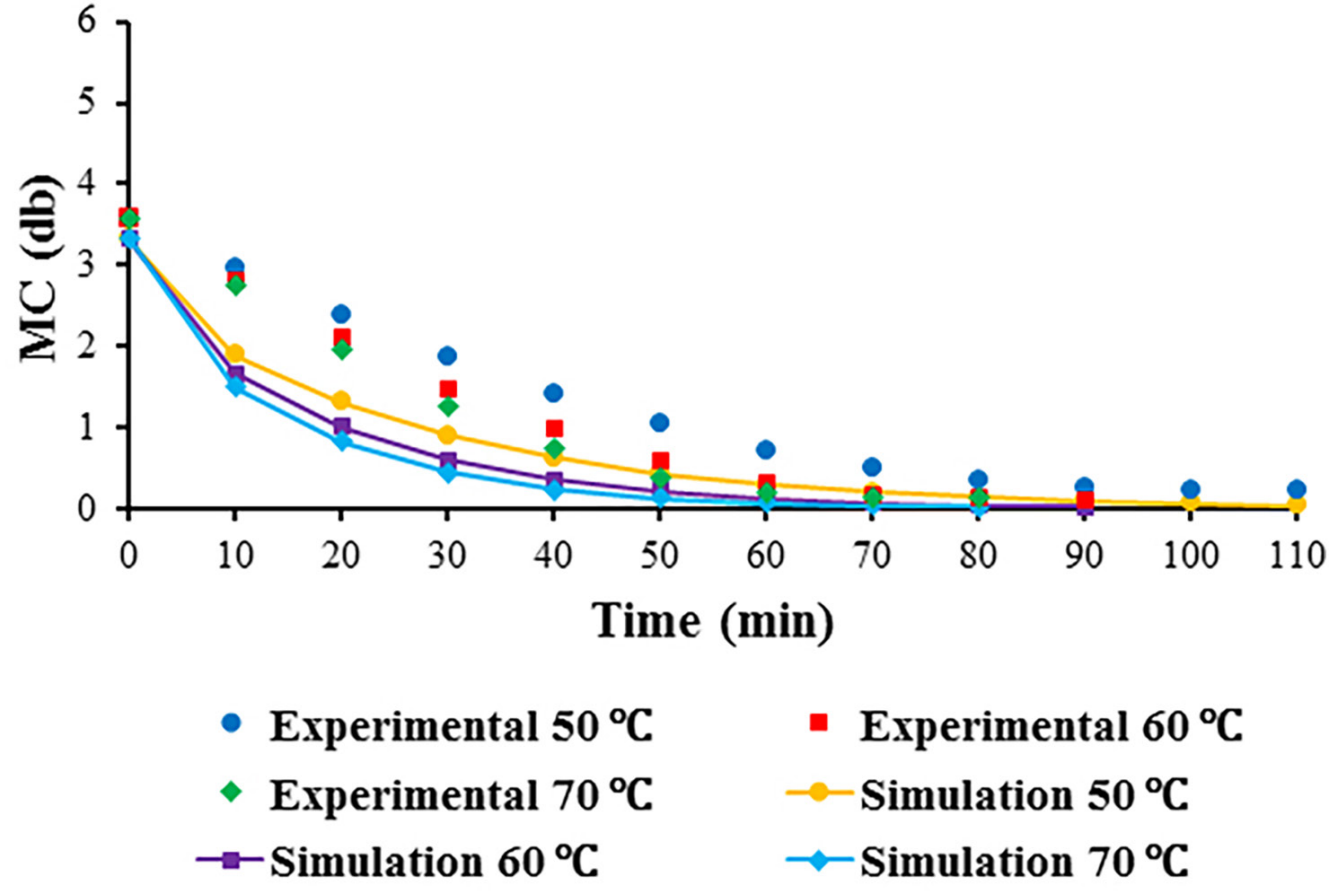
Moisture content comparison curves (experimental and simulation) at different drying temperatures (50, 60, and 70°C).

The comparison between experimental and predicted moisture content data at various temperatures is illustrated in Figure 13. The shorter drying duration to reach the intended moisture content was recorded at 70°C. Applying an elevated temperature during drying decreases relative humidity of the air, leading to the enhancement of water vapor pressure gradient between air and foam, thereby increasing drying rate and decreasing drying time (Do Nascimento et al., 2016).

**FIGURE 14:**
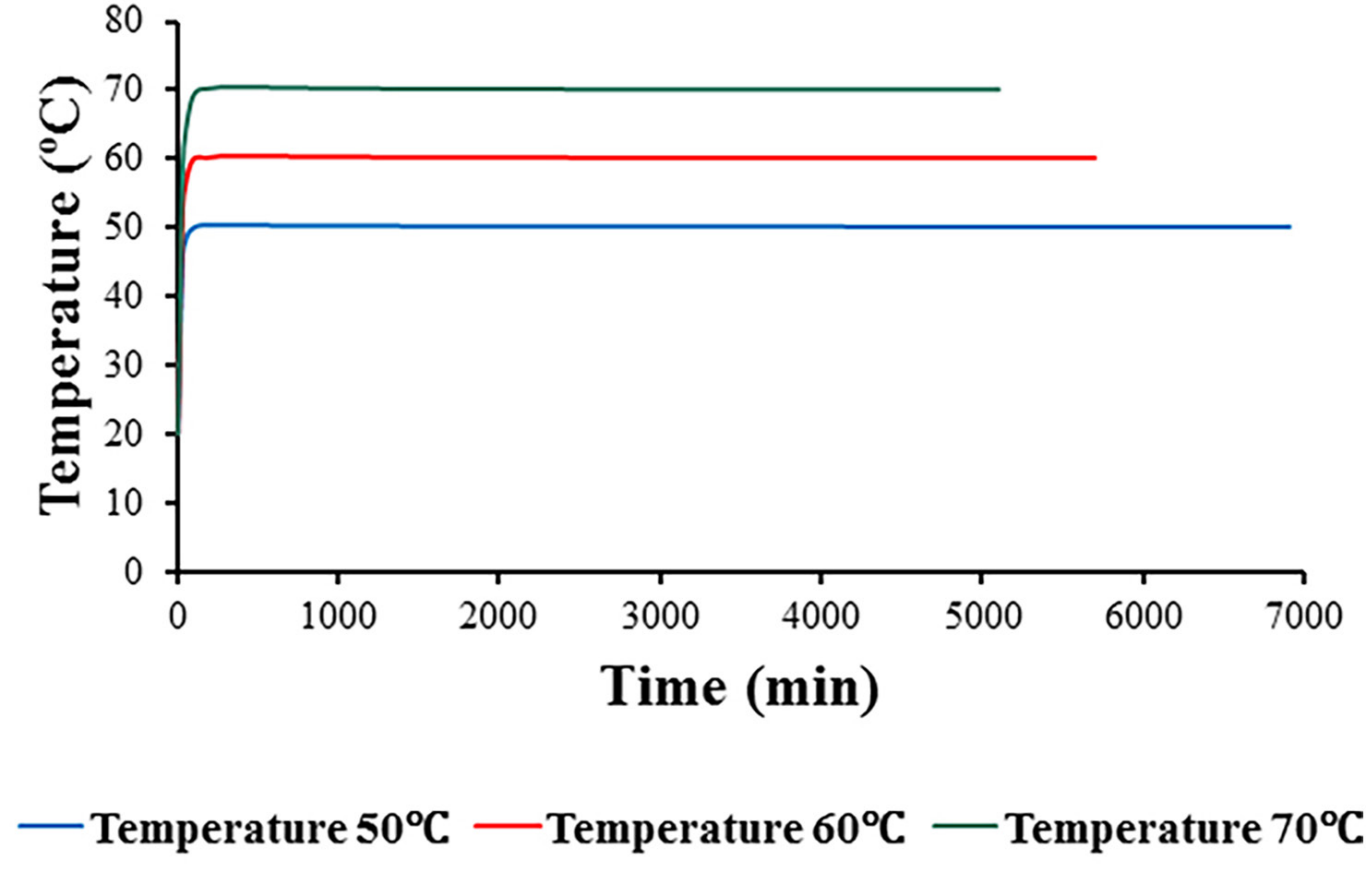
Average temperature profiles at various drying temperatures.

**FIGURE 15:**
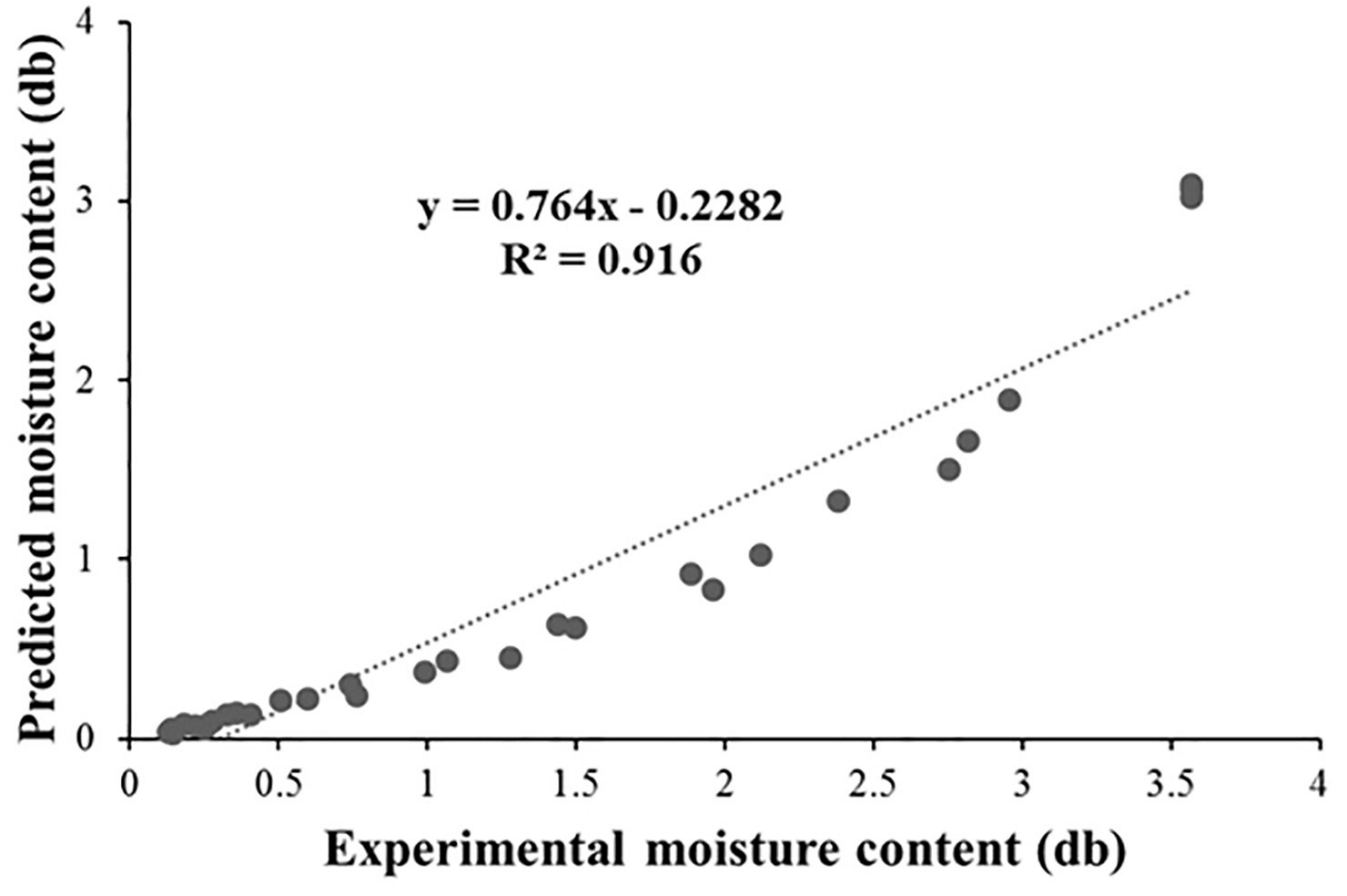
Predicted moisture content obtained from simulation vs. experimental moisture content of red beet pulp during foam‐mat drying at 50, 60, and 70°C.

Average temperature profiles showed that the temperature of foams reached a fixed value (temperature of air) at the earliest moments of drying (Figure 14). The average quantities of the foam temperature, according to the values obtained from simulation, were 50.03, 60.01, and 69.98 at air temperatures of 50, 60, and 70°C, respectively. Figure 15 shows model efficiency based on a regression graph of predicted vs. experimental moisture contents at all drying temperatures (50, 60, and 70°C). As can be seen in Figure 15, there was a good agreement between the simulated and empirical results with a correlation coefficient of 0.916 and a mean absolute error (MAE) of 0.538 (db). This difference could be considered satisfactory regarding different simulation and experimental parameters such as possible changes in experimental circumstances and thermophysical characteristics of the foams, as well as numerical fluctuations and model assumptions, which affect the model efficiency (Dehghannya et al., 2018).

## 5 CONCLUSIONS

Organic red pigments due to their coloring capacity, antioxidant, antimicrobial, and antitumor properties are considered safe replacements for synthetic red colorants in food industry. In this study, experimental and numerical investigations during foam‐mat drying of red beetroot were performed and the influence of various drying temperatures on physicochemical and microstructural qualities of the produced powder was examined. Results indicated that an increase in drying temperature brought about a significant increase in effective moisture diffusivity and drying rate. Moisture loss during foam‐mat drying of red beetroot happened in the falling‐rate period. As the drying temperature went up from 50 to 70°C, flowability of powders increased but water solubility and absorption indices were not influenced. Images obtained from field emission scanning electron microscope demonstrated that with raising drying temperature, wrinkling, cracking, and roughness of the powder particles were reduced. Moreover, computer simulation was carried out to evaluate the impact of hot air temperature on uniformity of heat and moisture profiles inside the foams during drying. Experimental validation of model showed a good agreement with predicted data. The developed methodology in this study could be used to control the foam‐mat drying process, produce various food powders, and enhance quality of the dried products. Further modeling improvements are required to increase the uniformity of the temperature and moisture profiles during the drying process.

## AUTHOR CONTRIBUTIONS

**Golnaz Bahriye:** Data curation (lead); formal analysis (lead); investigation (lead); methodology (lead); software (lead); visualization (lead); writing – original draft (lead). **Saeed Dadashi:** Conceptualization (equal); funding acquisition (equal); resources (equal); supervision (equal); validation (equal); visualization (equal); writing – review and editing (equal). **Jalal Dehghannya:** Conceptualization (equal); funding acquisition (equal); project administration (lead); resources (equal); software (lead); supervision (equal); validation (equal); visualization (equal); writing – review and editing (equal). **Hossein Ghaffari:** Resources (equal); visualization (equal).

## CONFLICT OF INTEREST STATEMENT

The authors declare that there is no conflict of interest.

## Data Availability

All data generated or analyzed during this study are included in this manuscript.
